# Book Review of “Biomaterials in Orthopaedics and Trauma: Current Status and Future Trends in Revolutionizing Patient Care”

**DOI:** 10.1007/s43465-025-01446-x

**Published:** 2025-06-16

**Authors:** Filippo Migliorini

**Affiliations:** 1 Department of Trauma and Reconstructive Surgery, University Hospital of Halle, Martin-Luther University Halle-Wittenberg, Ernst-Grube-Street 40, 06097 Halle (Saale), Germany; 2 Department of Orthopaedic and Trauma Surgery, Academic Hospital of Bolzano (SABES-ASDAA), via Lorenz Böhler 5, 39100 Bolzano, Italy; 3 Department of Life Sciences, Health, and Health Professions, Link Campus University, Via del Casale di San Pio V, 00165 Rome, Italy

**Fig. 1:**
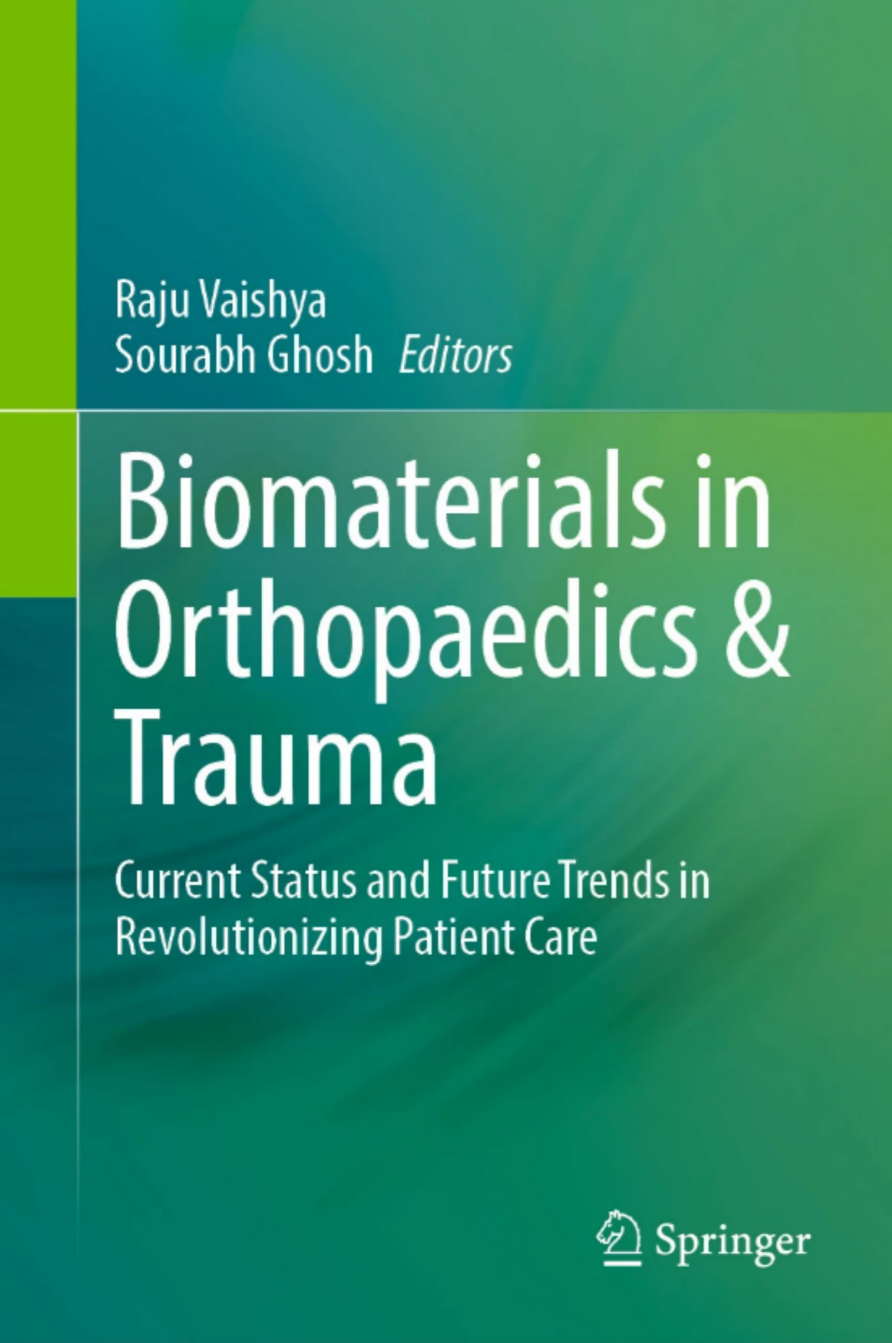
Cover page of the book

The inaugural edition of “*Biomaterials in Orthopaedics and Trauma: Current Status and Future Trends*” (https://link.springer.com/book/10.1007/978-981-96-3017-2), co-edited by the distinguished orthopaedic surgeon Dr. (Prof.) Raju Vaishya, who is ranked among the top 2% globally, and Prof. Sourabh Ghosh, a notable mechanical engineer, presents an exceptional compendium of contemporary insights into the integration of biomaterials within orthopaedic trauma care. Comprising 491 pages of meticulously curated content, this volume is more than just a publication; it represents a significant contribution to the field, inviting readers to explore the multifaceted applications of biomaterials in clinical practice. The title encapsulates the essence of the work, reflecting a dual focus on both current methodologies and the transformative trajectory of patient care through biomaterials. Enhanced by 71 colour illustrations and six black-and-white images, the book aids in a thorough understanding of this rapidly evolving domain. The structure is thoughtfully segmented into five principal sections, thoroughly addressing the comprehensive spectrum of biomaterials science. It explores pivotal research areas and their practical implications, highlighting revolutionary technologies such as 3D printing and additive manufacturing, stem cells, tissue engineering, sensor technology, and smart implants. Each section is designed to elucidate the significant advancements within these fields, thus providing the reader with a holistic perspective on the current landscape of biomaterials in orthopaedics. The chapters are carefully organised to demonstrate a coherent advancement in understanding. The introductory chapter thoroughly surveys the evolution and application of biomaterials within orthopaedics, effectively grounding readers in foundational concepts. The chapter dedicated to 3D printing and additive manufacturing (AM) elucidates how these advanced technologies have reshaped orthopaedic surgery, presenting empirical data on their impact and potential future applications. The discourse on stem cells and tissue engineering provides critical insights into their synergy with biomaterials, particularly in the context of innovative tissue repair methodologies. The subsequent chapters extend the discourse to practical applications in various orthopaedic procedures, with emphasis on joint replacements and the impact of modern biomaterials on surgical outcomes. With the global joint replacement market expected to reach $23.44 billion by 2026, the discussions surrounding sensor technology, smart implants, and nanotechnology highlight significant advancements that could dramatically enhance patient care and outcomes. This book distinguishes itself through an emphasis on interdisciplinary collaboration, merging insights from leading experts in research, clinical practice, and engineering across ten countries. With contributions from 75 expert authors, the book offers a rich and diverse perspective that not only covers the evolution and current applications of biomaterials but also inspires novel thinking regarding their potential future roles in musculoskeletal (MSK) healthcare. Designed to cater to a varied audience, the chapters are meticulously organised and presented with clarity, ensuring accessibility for both novices and seasoned professionals alike. The editors have skillfully identified emerging trends and future directions, fostering creative dialogue around biomaterials and their implications for improving patient outcomes. “Biomaterials in Orthopaedics and Trauma: Current Status and Future Trends in Revolutionizing Patient Care” is a commendable resource that presents a comprehensive overview of the evolving field of biomaterials. It should serve as an authoritative reference for students, clinicians, researchers, and academicians, providing invaluable insights into advancements in biomaterials and their pivotal roles in enhancing patient care outcomes (Fig. 1).

